# Investigation of auranofin and gold-containing analogues antibacterial activity against multidrug-resistant *Neisseria gonorrhoeae*

**DOI:** 10.1038/s41598-020-62696-3

**Published:** 2020-03-27

**Authors:** Ahmed Elkashif, Mohamed N. Seleem

**Affiliations:** 1 Department of Comparative Pathobiology, College of Veterinary Medicine, Purdue University, West Lafayette, IN 47907 USA; 2 Purdue Institute of Inflammation, Immunology, and Infectious Disease, West Lafayette, IN 47907 USA

**Keywords:** Drug discovery, Microbiology, Medical research

## Abstract

*Neisseria gonorrhoeae* represents an urgent public health threat due to the rapid emergence of resistance to current antibiotics and the limited number of anti-gonococcal agents currently in clinical trials. This study utilized a drug repositioning strategy to investigate FDA-approved gold-containing drugs against *N. gonorrhoeae*. Auranofin, sodium aurothiomalate and aurothioglucose inhibited 48 clinical isolates of *N. gonorrhoeae* including multidrug-resistant strains at a concentration as low as 0.03 µg/mL. A time-kill assay revealed that auranofin exhibited rapid bactericidal activity against *N. gonorrhoeae*. Moreover, both sodium aurothiomalate and aurothioglucose did not inhibit growth of vaginal protective commensal lactobacilli. Auranofin, in combination with azithromycin, ceftriaxone, cefixime or tetracycline showed an additive effect against four *N. gonorrhoeae* strains, suggesting the possibility of using auranofin in dual therapy. Moreover, auranofin reduced the burden of intracellular *N. gonorrhoeae* by over 99% outperforming the drug of choice ceftriaxone. Auranofin was found superior to ceftriaxone in reducing the secretion of the pro-inflammatory cytokine IL-8 by endocervical cells infected with *N. gonorrhoeae*. Furthermore, auranofin exhibited a prolonged post-antibiotic effect over 10 h, as well as inability to generate resistant mutants. Overall, the current study suggests that repurposing gold-containing drugs, like auranofin, for treatment of gonorrhea warrants further investigation.

## Introduction

*Neisseria gonorrhoeae* infects both the human male and female reproductive tracts causing the sexually transmitted infection gonorrhea. Gonorrhea is the second most common notifiable disease in the United States of America, according to the Centers for Disease Control and Prevention (CDC)^1,2^. The CDC estimates the number of new gonorrhea cases in the U.S. alone will exceed 820,000 cases annually, and this number is expected to increase due to extensive drug resistance^1^. Globally, the World Health Organization estimates 106 million new cases of gonorrhea will occur each year^2^. It has been reported that about 80% of *N. gonorrhoeae* cervical infections are mostly asymptomatic and unnoticed^3,4^. Without proper treatment, these infections can result in pelvic inflammation-associated damage to the ciliated epithelium^5^. This damage is permanent and can increase the risk of ectopic pregnancy and lead to tubal-factor infertility^6^.

With no available vaccines against *N. gonorrhoeae*, antibiotics are the only effective method to treat gonorrhea. Different countries have established different guidelines to treat gonorrhea, mostly comprised of dual therapy of a 1 g oral dose of azithromycin and an injectable 250 mg dose of intramuscular ceftriaxone for patients afflicted with gonorrhea^7–10^. However, increasing resistance to this dual therapy options and to the to the last available first-line treatments for gonorrhea^11,12^, increased the risk of untreatable gonorrhea becoming a widespread public health epidemic^12,13^. Thus, there is an urgent need for novel therapeutic strategies to treat gonorrhea.

In order to circumvent the cost and time associated with traditional *de novo* drug discovery, drug repositioning of FDA-approved drugs, particularly those that are off-patent, represents a promising approach to find new antibacterials^14^. Utilizing this strategy, we investigated auranofin as a potential novel antigonorrheal agent. Auranofin is an FDA-approved, gold-containing, drug used to treat rheumatoid arthritis. Previous studies on auranofin have reported that the drug possesses potent antibacterial activity against clinically-pertinent pathogens including methicillin-resistant *Staphylococcus aureus* (MRSA), vancomycin-resistant enterococci (VRE), and *Clostridioides difficile*^15–20^. However, no reports have investigated the effect of auranofin against *N. gonorrhoeae*. In this study, we investigated the three FDA approved compounds auranofin, aurothioglucose and sodium aurothiomalate, against a diverse panel of multidrug-resistant *N. gonorrhoeae* and against isolates of *Lactobacillus* that limit gonococcal colonization of the genitourinary tract. Additionally, the possibility of using auranofin in combination with antibiotics currently used to treat gonorrhea was explored. Furthermore, the gold compounds’ ability to reduce the burden of intracellular *N. gonorrhoeae* as well as its immunomodulatory effect were examined in an endocervical cell line infected with *N. gonorrhoeae*. Finally, we investigated the post-antibiotic effect of auranofin as well as its frequency of spontaneous resistance mutations.

## Results

### Susceptibility analysis of gold compounds against clinical isolates of *N. gonorrhoeae*

The anti-gonococcal activity of auranofin, sodium aurothiomalate and aurothioglucose against a panel of *N. gonorrhoeae* clinical isolates were determined using the broth microdilution assay. In addition, 5 WHO reference strains with well-characterized antibiogram, phenotypic and genetic markers^21^ were included to validate the results. As presented in Supplementary Table S2 the activity of control antibiotics tested via broth microdilution is almost identical to the values established by the WHO. The gold drugs were equipotent to or superior to azithromycin against the 5 reference strains as well as 48 other *N. gonorrhoeae* clinical isolates. As presented in Supplementary Table S3, auranofin inhibited growth of all *N. gonorrhoeae* strains tested at concentrations ranging from 0.007 µg/mL up to 0.125 µg/mL with MIC_50_ and MIC_90_ values of 0.06 µg/mL and 0.125 µg/mL, respectively (Table 1). Sodium aurothiomalate and aurothioglucose were slightly less potent compared to auranofin with MIC_50_ values of 0.25 µg/mL and 0.5 µg/mL respectively, and MIC_90_ values of 1 µg/mL and 8 µg/mL respectively (Table 1). Azithromycin’s MIC values ranged from 0.25 to 1 µg/mL against *N. gonorrhoeae* strains sensitive to this antibiotic while against azithromycin-resistant isolates (strains 167, 175, 179, 181, 197), the MIC of azithromycin ranged from 8 to 256 µg/mL(Supplementary Table S2). All three gold drugs maintained their effectiveness against *N. gonorrhoeae* clinical isolates exhibiting resistance to standard antibiotics used for treatment of gonorrhea such as *N. gonorrhoeae* strain 181 and *N. gonorrhoeae* strain 175, which are azithromycin-resistant strains. A group of 5 *N. gonorrhoeae* strains (167, 175, 179, 181, and 194) has been chosen for its high resistance profile to first line treatment for a confirmatory agar dilution according to the CLSI recommended agar dilution assay. There was no change observed in the MIC values obtained from either method. As depicted (Table 2), the MIC values of gold compounds and control antibiotics remained constant.

**Table 1:** The minimum inhibitory concentration 50 and 90 (MIC in µg/mL) of gold drugs (auranofin, sodium aurothiomalate and aurothioglucose) and control antibiotics azithromycin, and ceftriaxone against 48 clinical isolates of *Neisseria gonorrhoeae*. MIC_50_ and MIC_90_ are the minimum inhibitory concentrations needed to inhibit 50% and 90% of the strains.

Strain Name	Auranofin	Aurothiomalate	Aurothioglucose	Azithromycin	Ceftriaxone
MIC_50_	0.06	0.25	0.5	1	0.03
MIC_90_	0.125	1	8	4	0.06

**Table 2:** The minimum inhibitory concentration (MIC in µg/mL) of gold drugs (auranofin, sodium aurothiomalate and aurothioglucose) and control antibiotic azithromycin against four strains of *N. gonorrhoeae* (167, 175, 179, 181, and 197) via agar dilution.

Strain	Auranofin	Aurothiomalate	Aurothioglucose	Azithromycin
*N. gonorrhoeae 167*	0.125	0.5	8	16
*N. gonorrhoeae 175*	0.125	1	2	0.5
*N. gonorrhoeae 179*	0.06	0.125	0.25	**4**
*N. gonorrhoeae 181*	0.125	1	0.5	**265**
*N. gonorrhoeae 197*	0.06	0.5	0.5	8

### Evaluation of the gold drugs’ antibacterial activity against *N. gonorrhoeae* via a time-kill assay

**Figure 1:**
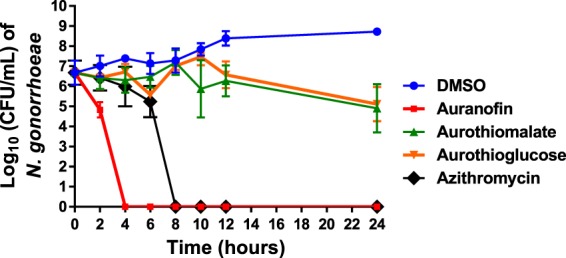
Time-kill analysis of gold compounds (at 3 × MIC) against *N. gonorrhoeae* strain 194 over a 24 hour incubation period at 37 °C. DMSO served as a negative control. Error bars represent standard deviation values. Lowest limit of detection is 100 CFU/mL.

After confirming all three gold-containing drugs possessed potent antibacterial activity against *N. gonorrhoeae*, we examined whether the three agents exhibit bacteriostatic or bactericidal activity. Auranofin, sodium aurothiomalate, aurothioglucose and azithromycin (all tested at 3 × MIC) were evaluated against *N. gonorrhoeae* strain 194 via a standard time-kill assay. As presented in Fig. 1, auranofin exhibited rapid bactericidal activity and completely eradicated the high bacterial inoculum within four hours. Azithromycin required eight hours to achieve the same effect. No reduction in bacterial inoculum was observed in the presence of both sodium aurothiomalate and aurothioglucose, at 3 × MIC, over 24 hours indicating these agents are bacteriostatic *in vitro* against *N. gonorrhoeae*.

### Combination testing of auranofin with control antibiotics against *N. gonorrhoeae*

**Figure 2:**
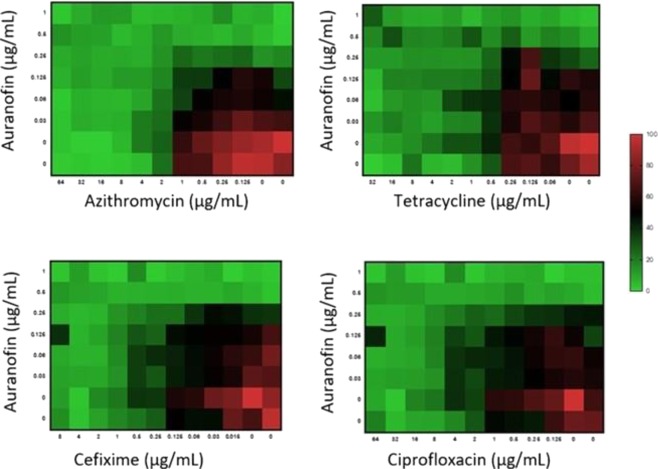
Checkerboard analysis of auranofin tested in combination with four different antibiotics against *N. gonorrhoeae* strain 194. Data presented as a heat map indicating additive relationship between auranofin, azithromycin, cefixime, ciprofloxacin or tetracycline (fractional inhibitory concentration index ranged from 1.25–2).

Antimicrobial resistance to gonorrhea treatment is continuously increasing; this necessitates new antibacterials and drug combinations be identified^22,23^. The current CDC guidelines for treatment of *N. gonorrhoeae* infections recommends dual therapy with two different antibiotics. The recommended treatment consists of a single dose of 250 mg of intramuscular ceftriaxone and 1 g of oral azithromycin in order to curb resistance forming to either agent. Given that auranofin exhibited the most potent *in vitro* antibacterial activity against *N. gonorrhoeae*, we investigated auranofin in combination with first-line antibiotics (azithromycin, ceftriaxone and cefixime) used to treat gonorrhea as well as tetracycline. Auranofin exhibited an additive relationship with all four drugs (fractional inhibitory concentration (FIC) index ranged from 1.2 to 2) against *N. gonorrhoeae* strain 194 (Fig. 2). This suggests that dual therapy using auranofin in combination with azithromycin, cefixime, ceftriaxone or tetracycline may be possible but requires further investigation.

### Selectivity of gold compounds to *N. gonorrhoeae* over commensal strains

*N. gonorrhoeae* pathogenesis greatly depends on colonization of the reproductive tract. One of the natural barriers of such colonization is an intact healthy microbiome. Species of lactobacilli present in the genitourinary tract play a significant role in hindering colonization by *Neisseria*. Thus, we explored the antibacterial activity of the gold compounds against eight commensal vaginal lactobacilli strains. As presented in Table 3, both sodium aurothiomalate and aurothioglucose were inactive against all eight strains of lactobacilli (MIC > 256 μg /mL), suggesting that they could be used against gonococci without disrupting the normal microbiota. Conversely, azithromycin, the drug of choice, inhibited the tested normal microbiota strains (MIC < 1 µg/mL). This was similar to the result obtained for auranofin (MIC values <1 µg/mL).

**Table 3:** The minimum inhibitory concentration (MIC in µg/mL) of gold drugs (auranofin, sodium aurothiomalate and aurothioglucose) and control antibiotic azithromycin against eight different commensal *Lactobacillus* strains from the female reproductive tract.

Strain	Auranofin	Aurothiomalate	Aurothioglucose	Azithromycin
*L. crispatus* HM638	<1	>128	>128	<1
*L. jensenii* HM105	<1	>128	>128	<1
*L. gasseri* HM-403	<1	>128	>128	<1
*L. johnsonii* HM643	<1	>128	>128	<1
*L. gasseri* HM642	<1	>128	>128	<1
*L. jensenii* HM639	<1	>128	>128	<1
*L. jensenii HM640*	<1	>128	>128	<1
*L. gasseri HM644*	1	>128	>128	<1

### Intracellular infection clearance assay

**Figure 3:**
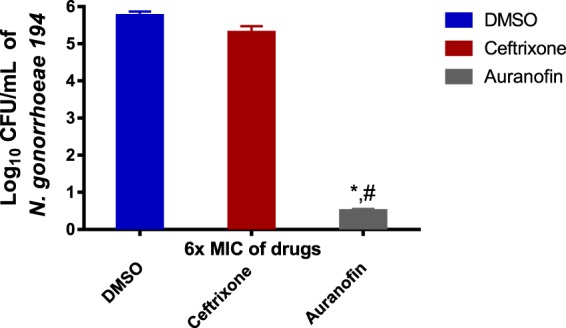
Effectiveness of auranofin and ceftriaxone (both at 6 × MIC) against intracellular *N. gonorrhoeae* in infected human endocervical cells (End1/E6E7). End1/E6E7 cells were infected with *N. gonorrhea* strain 194 for six hours and then treated with either auranofin or ceftriaxone for 24 hours. End1/E6E7 cells were subsequently lysed and intracellular bacterial CFU was determined. Error bars represent standard deviation values from triplicate samples used for each test agent. Auranofin was compared to both untreated cells (*) and cells treated with ceftriaxone (#). (P  < 0.01 analyzed via unpaired t-test).

*N. gonorrhoeae* has the ability to invade mucosal epithelia and survive intracellularly. An intracellular clearance assay was performed to determine if auranofin can clear intracellular *N. gonorrhoeae*. As depicted in Fig. 3 auranofin (at 6 × MIC) could successfully reduce the burden of intracellular *N. gonorrhoeae* infection of endocervical (END1/E6E7) by over 99% after 24 hours relative to the negative control. The control drug ceftriaxone could only achieve a non-significant reduction in intracellular bacterial counts. These results indicate that auranofin is able to effectively curb intracellular *N. gonorrhoeae* from infected endocervical cells.

### Auranofin reduces IL-8 production by endocervical cells infected with *N. gonorrhoeae*

**Figure 4:**
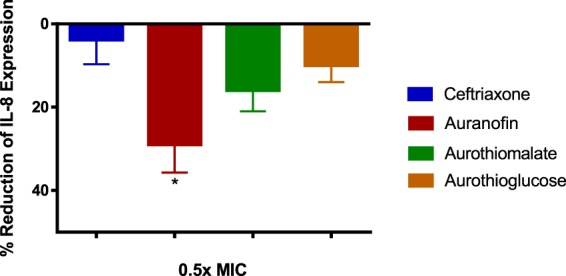
Evaluation of anti-inflammatory activity of gold drugs and ceftriaxone (all at 0.5 × MIC) in endocervical cells infected with *N. gonorrhoeae* strain 194. Auranofin and ceftriaxone reduced the level of IL-8 produced by End1/E6E7 cells 6 h post-infection. Error bars represent mean IL-8 measurement ± SD in three technical replicate wells from the same representative experiment. Significance indicated by (*) denotes a significant difference between cells treated with auranofin compared to ceftriaxone (P < 0.01 analyzed via One-way ANOVA).

Pro-inflammatory cytokines production is one of the hallmarks of *N. gonorrhoeae* infection. IL-8 is produced at the site of gonococcal infection and is elevated after the onset of symptoms and returns to baseline values after antibiotic therapy. Therefore, IL-8 is one of the inflammatory cytokines that mediates *N. gonorrhoeae* infection^24^. Auranofin is known to possess potent anti-inflammatory activity but its effect on IL-8 in endocervical cells infected with *N. gonorrhoeae* has not been previously reported. Thus we determined the amount of IL-8 produced from *N. gonorrhoeae* 194 infected End1/E6E7 cells, in the presence or absence of gold compounds and the control antibiotic ceftriaxone (at ½ × MIC). DMSO was used as a negative control to determine the baseline amount of IL-8 produced by infected endocervical cells. Auranofin was able to significantly reduce the level of IL-8 produced by infected endocervical cells by 28% relative to DMSO (Fig. 4). This was superior to ceftriaxone, which only achieved a 4.1% reduction. Though both sodium aurothiomalate and aurothioglucose reduced IL-8 production from infected endocervical cells by more than 10%, it was not statistically different compared to ceftriaxone treatment.

### Frequency of spontaneous mutation

Given the promising *in vitro* results of auranofin described above we next sought to investigate the likelihood that *N. gonorrhoeae* will develop resistance to auranofin using the single step resistance assay method^19^ and the results are reported in Supplementary Table S4. No resistant mutants were isolated at a concentration of 10 × MIC in the presence of a high inoculum, equal to 2.4 × 10^10^ CFU/mL, of *N. gonorrhoeae* strains 197, 202 or 206; indicating a resistance frequency < 2.4 × 10^−10^ for all tested strains which was lower than the drug of choice azithromycin. The positive control, rifmapicin’s frequency of spontaneous mutation was 1.8 ×10^−6^ which is equal to the value previously reported in other bacterial strains^25,26^.

### Post-antibiotic effect

The ability of auranofin to exhibit a prolonged inhibitory effect against *N. gonorrhoeae* following a brief exposure period was investigated through analyzing its post-antibiotic effect (PAE). Following a 2-hour exposure to 10 × MIC of either auranofin or azithromycin, four strains of *N. gonorrhoeae* were allowed to grow for 12 hours, sampled and plated every 2 hours. The results of the experiment were analyzed and reported in Supplementary Table S5. Auranofin exhibited a long PAE against all tested strains as it inhibited their growth for 10 hours post exposure. This was superior to the drug of choice azithromycin which showed only PAE = 8 hours.

## Discussion

The timeline of antibiotic development against *N. gonorrhoeae* has always been paralleled by rapid resistance acquisition by the bacterium. Since the development of sulfonamides in the 1930s, *N. gonorrhoeae* has acquired resistance to every first-line antibiotic class introduced^27^. More recently, the problem has worsened with the emergence of new bacterial strains exhibiting multidrug-resistant and extensively drug-resistant phenotypes. The emergence of untreatable strains of *N. gonorrhoeae* combined with the limited pipeline of novel antigonococcal agents underscores the critical need to discover new antibacterial agents^28,29^. Revisiting FDA-approved drugs for an alternative indication to treat gonorrhea can be a fast and effective strategy to provide new therapeutic options or identify molecules with weak antibacterial activity that can be modified to enhance their potency. Using a drug-repositioning approach, we identified auranofin and two gold thiol drugs, aurothioglucose and aurothiomalate, as potential candidates for investigation to treat drug-resistant gonorrhea.

The antibacterial activity of auranofin and its analogues was explored using broth microdilution method, which was herein validated using five *N. gonorrhoeae* strains from WHO used for quality assurance and quality control of gonococcal AMR testing. A panel of 48 *N. gonorrhoeae* clinical isolates was tested and to further confirm the method 5 strains with different resistance profile to control antibiotics were selected for a run via agar dilution yielded the same results. Auranofin and its two analogues successfully inhibited the growth of all the tested isolates of *N. gonorrhoeae*. Auranofin was the most potent drug against *N. gonorrhoeae* with MIC values ranging from 0.03 to 0.25 µg/mL. Aurothiomalate’s MIC values ranged from 0.06 to 32 µg/mL while aurothioglucose’s MIC values ranged from 0.125 to 16 µg/mL. In a time-kill assay, auranofin (at 3 × MIC) showed rapid bactericidal activity against *N. gonorrhoeae*, successfully reducing the bacterial count below the limit of detection (250 CFU/mL) after only four hours. Being the most potent of the tested gold compounds, auranofin was chosen for further analysis. Moreover, to address the problem of rapid emergence of resistance of *N. gonorrhoeae* to auranofin we examined the possibility of isolating auranofin resistant mutants after carrying out a single step resistance assay. At a high inoculum size (~10^10^ CFU/mL), no mutants were obtained indicating that spontaneous resistance mutations of *N. gonorrhoeae* against auranofin did not occur at a high inoculum, on the other hand resistance was developed against rifampicin which is in agreement with a previous report in *S. aureus*^19^. However, other long term resistance studies should be performed to further investigate the potential of resistance development against auranofin.

Pharmacodynamic parameters such as the post antibiotic effect are increasingly applied to help shape a dosing regimen (size and frequency of deses given to a patient) for antibiotics. The post-antibiotic effect is defined as the period through which bacterial growth will be suppressed after a brief exposure to an antibiotic^30^. The post-antibiotic effect analysis performed provides valuable evidence that auranofin would need fewer doses if used to treat *N. gonorrhoeae* infections. As auranofin exhibited a long PAE (=10 hours) against *N. gonorrhoeae*, this indicates a very slow bacterial recovery after exposure and hence patients would need to be subjected to fewer doses, which is advantageous in terms of limited toxicity to host tissues, reduced costs, and more patient compliance to the prescribed regimen^31^.

The presence of a healthy microbiome in the female reproductive tract can aid in reducing susceptibility to sexually transmitted diseases^32^. In particular, vaginal species of lactobacilli in women can reduce the risk of contracting infections from *G. vaginalis* and *N. gonorrhoeae*^33^. In addition, women whose vaginal microbiota are most dominated by lactobacilli species have a lower incidence of *N. gonorrhoeae* infection^34,35^. Previous studies have shown that dysbiosis contributes to gonococcal infections. However, first-line gonorrhoeae treatments unfortunately inhibit growth of important bacterial species that comprise the vaginal microbiome and hinder colonization by gonococci. Finding alternative therapeutics that selectively target gonococci without inhibiting important commensal species such as lactobacilli would be desirable. We thus investigated the antibacterial activity of the gold-containing drugs against important lactobacilli species that comprise the vaginal microbiome. Aurothioglucose and sodium aurothiomalate exhibited high selectivity towards *N. gonorrhoeae* while not inhibiting growth of eight different commensal lactobacilli strains. In contrast, auranofin exhibited similar potency against species of lactobacilli and *N. gonorrhoeae*, which was similar to azithromycin.

*N. gonorrhoeae* can invade the female genital tract epithelial cells and cross the epithelial barrier into the sub-epithelial space^36^. *N. gonorrhoeae* has been previously shown to be able to survive inside host cells as well as to pass epithelial cell layers, which is a key step in disseminated infections. Auranofin was able to clear *N. gonorrhoeae* infection *in vitro*, thus we investigated its ability to clear intracellular bacteria present in infected endocervical cells. After 24 hours, at 6 × MIC, auranofin was superior to the drug of choice ceftriaxone in reducing the burden of intracellular bacteria inside infected END1/E6E7 endocervical cells. Auranofin was able to achieve a 5.0 −log_10_ reduction in *N. gonorrhoeae* inside infected endocervical cells while ceftriaxone, on the contrary, was not significantly effective. To rule out the possibility of cell death leading to a false positive result, auranofin was assayed for its *in vitro* cytotoxicity against End1/E6E7 endocervical cells for 24 hours via MTS cytotoxicity assay^37^. As depicted in Supplementary Fig. S6, cells viability was around 78% and 85% at 8× and 4× MIC respectively. Collectively, these results indicate that auranofin can significantly clear intracellular *N. gonorrhoeae* at a rate outperforming the drug of choice ceftriaxone.

Successful colonization by *N. gonorrhea* of the female urogenital tract is accompanied by a severe inflammatory response that results in production of numerous pro-inflammatory cytokines^38^. Alleviating such inflammation is a potential approach to reduce the infection’s severity. It has been previously reported that gold drugs such as auranofin have potent anti-inflammatory activity and can reduce the expression of pro-inflammatory cytokines such as tumor necrosis factor-α, interleukin-6, interleukin-1 β, and monocyte chemoattractant protein-1^18^. Based upon this finding, we hypothesized the gold compounds investigated in this report would reduce the production of pro-inflammatory cytokines by endocervical cells infected with *N. gonorrhoeae*. In this regard, auranofin was able to significantly reduce IL-8 expression of pre-infected human endocervical cells. This effect was superior to the effect of the drug of choice ceftriaxone. The anti-inflammatory effect of auranofin could be advantageous to curbing damage of the epithelial tissue present in the urogenital tract in patients afflicted with gonorrhea.

It is important to mention that the data of the clinical trial of auranofin for treatment of rheumatoid arthritis which included over 5000 patients administered (6 mg/day) revealed no signs of accumulated toxicity and good tolerability^39^. In a recent clinical trial of auranofin, through a maximal daily dose of 21 mg over 14 days regimen, trough plasma concentrations of auranofin ranging between 0.8–1.5 mg/L after the 14^th^ day were measured with a half-life time of 35 days^40^. These pharmacokinetic properties will assure a higher concentration of auranofin than the MICs covered in this study. Moreover, the treatment course of rheumatoid arthritis can extend to over 5 years. Such treatment plan is much longer than the course of treatment usually prescribed for antibiotics which ranges from one to two weeks^29^. The toxicity of auranofin over HaCaT cells was evaluated to be 6.38 ± 0.29 μg/mL^18^. This value is 100 times greater than the MIC_50_ value of auranofin against *N. gonorrhoeae*.

To conclude, we report that three FDA-approved gold drugs, auranofin, aurothiomalate and aurothioglucose have potent *in vitro* antibacterial activity against *N. gonorrhoeae*. Aurothiomalate and aurothioglucose possess an advantage over current antibiotics used to treat gonorrhea in that both gold-containing drugs do not inhibit growth of commensal *Lactobacillus spp*. that provide a protective barrier to gonococcal colonization. Auranofin was found to possess advantageous properties than drugs of choice such as having an additive relationship with antibiotics used to treat gonorrhea, which may open the door for additional dual therapy treatment options for gonorrhea, a very low frequency of spontaneous mutation which suggests a low probability to develop resistance and a highly prolonged post-antibiotic effect that suggests smaller and less frequent doses of the drug if used to treat gonorrhea. Finally, auranofin was found to reduce the burden of intracellular *N. gonorrhoeae* infecting endocervical cells as well as modulate the production of the pro-inflammatory cytokine IL-8, which can be advantageous in reducing inflammation, one of the hallmarks of gonococcal infection. A future aim is to validate the *in vitro* results presented in this study *in vivo* via a rodent model of *N. gonorrhoeae* infection.

## Materials and Methods

### Bacterial strains, chemicals and media

The *N. gonorrhoeae* reference strains were obtained from the WHO reference strain panel for global quality assurance and quality control of gonococcal AMR testing^21,41^. The rest of *N. gonorrhoeae* strains used were clinical isolates obtained from the CDC (Supplementary Table 1). The commensal vaginal bacterial strains were obtained from the Biodefense and Emerging Infections Research Resources Repository (BEI Resources) (Supplementary Table 2). All chemical compounds used in this study were obtained commercially from chemical vendors: auranofin (Chem-Impex International, Wood Dale, IL), sodium aurothiomalate, tetracycline and ciprofloxacin (Sigma-Aldrich, St. Louis, MO), aurothioglucose (USP reference standard Rockville, MD), ceftriaxone and cefixime (Acros Organics, NJ), and azithromycin (TCI America, Portland, OR).

Difco lactobacilli MRS broth, Brucella base broth and chocolate II agar (GC II Agar with hemoglobin and IsoVitaleX) were purchased from Becton, Dickinson and Company (Cockeysville, MD). Yeast extract and dextrose (Fisher Bioreagents, Fairlawn, NJ), proteose-peptone, nicotinamide adenine dinucleotide (NAD) and agarose (Sigma-Aldrich, St. Louis, MO), hematin solution, Tween 80, and pyridoxal (Chem-Impex International, Wood Dale, IL) were purchased from chemical vendors.

### Antibacterial susceptibility analysis for gold drugs and control antibiotics

The minimum inhibitory concentration (MIC) of gold drugs (auranofin, sodium aurothiomalate, and aurothioglucose) and azithromycin was determined using the broth microdilution assay, as previously described^42^. Briefly, a 1.0 McFarland standard was prepared and diluted in Brucella Supplemented broth (BSB) (Brucella broth supplemented with yeast extract, dextrose, agarose, proteose-peptone, NAD, pyrodixal and hematin) to reach a bacterial count of 5 × 10^5^ CFU/mL. Drugs were added and serially diluted in broth. Plates were then incubated for 24 hours at 37 °C in the presence of 5% CO_2_. In order to confirm the MIC values obtained by broth microdilution a confirmatory test was carried out on five *N. gonorrhoeae* strains (167, 175, 179, 181, 197), which have different resistance profile to azithromycin, ciprofloxacin and ceftriaxone, according to the CLSI recommended agar dilution as described^43^.

### Time-kill kinetics of gold compounds and azithromycin against *N. gonorrhoeae*

The killing kinetics of the gold drugs was investigated following the method described in a previous study^44^. In brief, an overnight culture of *N. gonorrhoeae* strain 194 was diluted in fresh BSB and incubated until the inoculum was ~5 × 10^6^ CFU/mL. The bacterial solution was subsequently exposed to 3 × MIC of the tested drugs (in triplicates). DMSO (Solvent for drugs) served as a negative control and azithromycin was used as a positive control. Aliquots were collected after 0, 2, 4, 6, 8, 10, 12, and 24 hours and plated onto GC II agar supplemented with hemoglobin and 1% IsoVitaleX. Plates were incubated at 37 °C with 5% CO_2_ for 24 hours prior to counting colonies present on the plate. The experiment was repeated 3 times with lowest limit of detection was 250 CFU/mL.

### Vaginal microbiome sensitivity to gold drugs

The selectivity of the three gold-containing drugs to pathogenic gonococci over bacterial species that comprise the vaginal commensal flora of the microbiome was assessed by determining the drugs’ MIC against *Lactobacillus gasseri, L. jensenii, L. rhamnosus*, and *L. crispatus*. Lactobacilli were streaked on de Man, Rogosa, Sharpe (MRS) agar and incubated for 48 hours at 37 °C with 5% CO_2._ The broth microdilution assay method was utilized to determine the MIC of the gold drugs and azithromycin following the guidelines outlined by the Clinical and Laboratory Standards Institute^43,45^.

### Synergistic activity of auranofin with azithromycin, cefixime, ciprofloxacin and tetracycline

The ability of auranofin to work in combination with conventional antibiotics used in the treatment of gonorrhea was evaluated as previously described^46,47^. In brief, a bacterial suspension of *N. gonorrhoeae* strain 194 equivalent to 1.0 McFarland standard was prepared and diluted in BSB to achieve a bacterial inoculum of 5 × 10^5^ CFU/mL. Subsequently, auranofin and control drugs were added at different concentrations along with bacteria containing media. The plates were incubated for 24 hours at 37 °C in the presence of 5% CO_2_. Next, the optical density was measured at 600 nm, using Spectra Max i3 (Molecular Devices, LLC, San Jose, CA) spectrophotometer, to calculate the percentage growth of bacteria. Results were plotted as a heat map and fractional inhibitory concentrations indices (FIC) were calculated. In this analysis, interactions with calculated FIC that were ≤0.5 were categorized as synergistic. An FICI of >0.5 but ≤1.25 was categorized as additive. An FICI > 1.25 but ≤4 was considered as indifference, while an FICI > 4 was categorized as antagonistic^16,17^.

### Intracellular clearance assay

In order to investigate the ability of auranofin to enter human endocervical cells and reduce the burden of intracellular *N. gonorrhoeae* an intracellular bacterial clearance assay was utilized as previously described^42^. Briefly, a cell density of ~1 ×10^5^ per well of human endocervical cells (ATCC CRL-2615, End1/E6E7) were seeded in 96-well tissue culture and then infected with *N. gonorrhoeae* strain194 at a multiplicity of infection of 100:1 for six hours at 37 °C and 5% CO_2_. Cells were then washed with 320 μg/mL gentamicin containing PBS to remove and wash-off non-phagocytized bacteria and then incubated with 32 μg/mL gentamicin containing PBS for 4 hours. Either auranofin or the control drug ceftriaxone were added subsequently to the cells at 6 × MIC and incubated for 24 hours at 37 °C with 5% CO_2_. The cells were lysed the following day and the plated onto Chocolate II Agar plates. Experiments were performed using triplicate samples for each treatment group and the experiment was repeated at least twice. Data were analyzed using an unpaired t-test using GraphPad Prism 6.00 (GraphPad Software, La Jolla, CA).

### Anti-inflammatory activity of gold-containing drugs on IL-8 expression by infected endocervical cells

To investigate the anti-inflammatory activity of the gold compounds, IL-8, a pro-inflammatory cytokine, was detected in the supernatants of *N. gonorrhoeae* infected End1/E6E7 – human endocervix cells (ATCC CRL-2615) exposed to either the gold-containing drugs or ceftriaxone, as previously described^42,48^. In brief, *N. gonorrhoeae* strain 194 was allowed to infect the endocervical cells for two hours at 37°C with 5% CO_2_ before initiating treatment with 0.5 × MIC of ceftriaxone, auranofin, sodium aurothiomalate, or aurothioglucose (tested in triplicates) and left for four hours. DMSO served as a negative control. Supernatants of cells were collected and tested for IL-8 concentration using the Human IL-8 ELISA Kit (Human IL-8 PicoKine ELISA Kit, Boster Biological Technology, Pleasanton, CA) following the manufacturer’s protocol. Data were analyzed using a one-way ANOVA with post-hoc Dunnett’s multiple comparisons test using GraphPad Prism 6.0 (GraphPad Software, La Jolla, CA).

### *N. gonorrhoeae* frequency of spontaneous mutation

Comprising a wide range of antibacterial susceptibility to control antibiotics, *N. gonorrhoeae* strains 197, 202 and 206 were chosen to be further investigated to determine the frequency of mutation. The bacteria were tested against both auranofin and rifampicin at 10× the MIC determined via agar dilution assay following the procedure that was previously described in^19^. Rifampicin was used as a positive control as it is known to be susceptible to spontaneous mutations^49,50^. Briefly, to prepare the media containing the drugs, GC agar base was autoclaved for 30 mins then human blood was added after cooling down to a final concentration of 5%. Either auranofin, or rifampin were then added to achieve a final concentration of 10 × MIC inside the media which was then poured in plates and left to dry out. A high inoculum ~1.0 × 10^10^CFU/mL of each *N. gonorrhoeae* strain tested was plated and incubated for 48 hours. Plates were checked after 48 hours to count grown colonies.

### Post-antibiotic effect of auranofin

For PAE testing the assay was carried out on four *N. gonorrhoeae* strains with various resistance profiles (strains 181,186,194,198) following the procedure previously described^51^. Briefly, 10 × MIC of either auranofin or control antibiotic azithromycin were added to tubes containing 2 mL of BSB inoculated with 1.0 × 10^6^ CFU/mL from each strain. Tubes were incubated in shaking incubator at 37 °C for one-hour exposure period after which cultures were diluted 1:1000 with pre-warmed BSB to remove the antibiotic. The tubes were then returned back to same incubation conditions. The tubes were routinely sampled and plated on chocolate agar plates before exposure and immediately after dilution every 2 hours for 12 hours. The PAE was calculated as follows: PAE = T − C, where T is the time taken by the viable counts of an antibiotic exposed culture to increase by 1 log_10_ unit above the count observed right after dilution, while C is the corresponding time for the growth of control.

## Supplementary information

Supporting Information.
