# Supplementary material for: Investigation of auranofin and gold-containing analogues antibacterial activity against multidrug-resistant Neisseria gonorrhoeae
Source: Sci Rep. 2020 Mar 27;10:5602. doi: 10.1038/s41598-020-62696-3 (PMC7101410; doi:10.1038/s41598-020-62696-3)
Supplement: Supplementary file 1 — Supporting Information. [file 41598_2020_62696_MOESM1_ESM.docx]

**Supplementary materials**

**Investigation of auranofin and gold-containing analogues antibacterial activity against multidrug-resistant Neisseria gonorrhoeae**

Ahmed Elkashif^1^and Mohamed N. Seleem^1,2*^

^1^ Department of Comparative Pathobiology, College of Veterinary Medicine, Purdue University, West Lafayette, IN, 47907, USA

^2^ Purdue Institute of Inflammation, Immunology, and Infectious Disease, West Lafayette, IN, 47907, USA

**Supplementary Table S1. Bacterial strains that were used in this study.**

| **Bacterial Strain** | **Description** |
| --- | --- |
| *N. gonorrhoeae* WHO F | (origin: Canada, 1991) |
| *N. gonorrhoeae* WHO G | Resistant to tetracycline, and ciprofloxacin (origin: Thailand, 1997) |
| *N. gonorrhoeae* WHO L | Resistant to tetracycline, penicillin, and ciprofloxacin (origin: Asia, 1996) |
| *N. gonorrhoeae* WHO M | Resistant to penicillin and ciprofloxacin (origin: Philippines, 1992) |
| *N. gonorrhoeae* WHO O | Resistant to penicillin (origin: Canada, 1991) |
| *N. gonorrhoeae* [*214*](https://wwwn.cdc.gov/ARIsolateBank/Panel/IsolateDetail.aspx?IsolateID=214) | Resistant to tetracycline, penicillin, and ciprofloxacin |
| *N. gonorrhoeae* [213](https://wwwn.cdc.gov/ARIsolateBank/Panel/IsolateDetail.aspx?IsolateID=213) | Resistant to tetracycline, *penicillin*, and ciprofloxacin |
| *N. gonorrhoeae* [211](https://wwwn.cdc.gov/ARIsolateBank/Panel/IsolateDetail.aspx?IsolateID=211) | Resistant to tetracycline, penicillin, and ciprofloxacin |
| *N. gonorrhoeae* [210](https://wwwn.cdc.gov/ARIsolateBank/Panel/IsolateDetail.aspx?IsolateID=210) | Resistant to tetracycline, penicillin, and ciprofloxacin |
| *N. gonorrhoeae* [209](https://wwwn.cdc.gov/ARIsolateBank/Panel/IsolateDetail.aspx?IsolateID=209) | Resistant to tetracycline, penicillin, and ciprofloxacin |
| *N. gonorrhoeae* [208](https://wwwn.cdc.gov/ARIsolateBank/Panel/IsolateDetail.aspx?IsolateID=208) | Resistant to tetracycline, penicillin, and ciprofloxacin |
| *N. gonorrhoeae* [207](https://wwwn.cdc.gov/ARIsolateBank/Panel/IsolateDetail.aspx?IsolateID=207) | Resistant to tetracycline, penicillin, and ciprofloxacin |
| *N. gonorrhoeae* [206](https://wwwn.cdc.gov/ARIsolateBank/Panel/IsolateDetail.aspx?IsolateID=206) | Resistant to tetracycline, penicillin, and ciprofloxacin |
| *N. gonorrhoeae* [205](https://wwwn.cdc.gov/ARIsolateBank/Panel/IsolateDetail.aspx?IsolateID=205) | Resistant to tetracycline, penicillin, and ciprofloxacin |
| *N. gonorrhoeae* [204](https://wwwn.cdc.gov/ARIsolateBank/Panel/IsolateDetail.aspx?IsolateID=204) | Resistant to tetracycline, penicillin, and ciprofloxacin |
| *N. gonorrhoeae* [203](https://wwwn.cdc.gov/ARIsolateBank/Panel/IsolateDetail.aspx?IsolateID=203) | Resistant to tetracycline, penicillin, and ciprofloxacin |
| *N. gonorrhoeae* 202 | Resistant to azithromycin |
| *N. gonorrhoeae* 201 | Resistant to tetracycline, penicillin, and ciprofloxacin |
| *N. gonorrhoeae* 200 | Resistant to tetracycline, penicillin, and ciprofloxacin |
| *N. gonorrhoeae* 199 | Resistant to tetracycline and penicillin |
| *N. gonorrhoeae* 198 | Resistant to tetracycline, penicillin, and ciprofloxacin |
| *N. gonorrhoeae* 197 | Resistant to tetracycline, penicillin, and ciprofloxacin |
| *N. gonorrhoeae* 196 | Resistant to tetracycline, penicillin, and ciprofloxacin |
| *N. gonorrhoeae* 195 | Resistant to tetracycline, penicillin, and ciprofloxacin |
| *N. gonorrhoeae* 194 | Resistant to penicillin, not susceptible to ceftriaxone, cefixime and cefpodoxime |
| *N. gonorrhoeae* 193 | Resistant to tetracycline and penicillin |
| *N. gonorrhoeae* 192 | Resistant to tetracycline, penicillin, and ciprofloxacin |
| *N. gonorrhoeae* 191 | Resistant to tetracycline, penicillin, and ciprofloxacin |
| *N. gonorrhoeae* 190 | Resistant to tetracycline, penicillin, and ciprofloxacin |
| *N. gonorrhoeae* 189 | Resistant to tetracycline, penicillin, and ciprofloxacin |
| *N. gonorrhoeae* 188 | Resistant to tetracycline, penicillin, and ciprofloxacin |
| *N. gonorrhoeae* 187 | Resistant to penicillin |
| *N. gonorrhoeae* 186 | Resistant to tetracycline, penicillin, and ciprofloxacin |
| *N. gonorrhoeae* 185 | Resistant to tetracycline, penicillin, and ciprofloxacin |
| *N. gonorrhoeae* 184 | Resistant to tetracycline, penicillin, and ciprofloxacin |
| *N. gonorrhoeae* 183 | Resistant to tetracycline, penicillin, and ciprofloxacin |
| *N. gonorrhoeae* 182 | Resistant to tetracycline, penicillin, and ciprofloxacin |
| *N. gonorrhoeae* 181 | Resistant to tetracycline and azithromycin |
| *N. gonorrhoeae* 180 | Resistant to tetracycline, penicillin, and ciprofloxacin |
| *N. gonorrhoeae* 179 | Resistant to azithromycin |
| *N. gonorrhoeae* 178 | Resistant to tetracycline, penicillin, and ciprofloxacin |
| *N. gonorrhoeae* 177 | Resistant to tetracycline |
| *N. gonorrhoeae* 176 | Resistant to tetracycline, penicillin, and ciprofloxacin |
| *N. gonorrhoeae* 175 | Resistant to azithromycin |
| *N. gonorrhoeae* 173 | Resistant to tetracycline, penicillin, and ciprofloxacin |
| *N. gonorrhoeae* 172 | Resistant to tetracycline, penicillin, and ciprofloxacin |
| *N. gonorrhoeae* 171 | Resistant to tetracycline, penicillin, and ciprofloxacin |
| *N. gonorrhoeae* 170 | Resistant to tetracycline, penicillin, and ciprofloxacin |
| *N. gonorrhoeae* 169 | Resistant to tetracycline, penicillin, and ciprofloxacin |
| *N. gonorrhoeae* 168 | Resistant to tetracycline, penicillin, and ciprofloxacin |
| *N. gonorrhoeae* 167 | Resistant to azithromycin |
| *N. gonorrhoeae* 166 | Resistant to tetracycline, penicillin, and ciprofloxacin |
| *N. gonorrhoeae* 165 | Resistant to tetracycline, penicillin, and ciprofloxacin |
| *L. gasseri* HM-642 | Vaginal isolate from a healthy US woman, obtained in 2007 |
| *L. gasseri* HM-644 | Vaginal mucosal isolate from a healthy US woman of child-bearing age, obtained in 2007 |
| *L. gasseri* HM-403 | Isolated from human patient’s mid-vaginal wall in March 2010 in Richmond, Virginia |
| *L. crispatus* HM-638 | Vaginal isolate from a healthy Chinese woman, obtained in 2007 |
| *L. jensenii* HM-640 | Isolated in 2007 from the vaginal mucosa of a healthy Chinese woman |
| *L. jensenii* HM-105 | Human vaginal isolate obtained from Texas |
| *L. jensenii* HM-639 | Isolated in 2007 from the vaginal mucosa of a healthy US woman |
| *L. johnsonii* HM-643 | Isolated in 2007 from the vaginal mucosa of a Chinese woman |

**Supplementary Table S2. The minimum inhibitory concentration (MIC in µg/mL) of control antibiotics azithromycin, ciprofloxacin, cifixime, gentamicin, tetracyclin and ceftriaxone against 5 *Neisseria gonorrhoeae* reference strains obtained from the WHO reference strain panel for global quality assurance and quality control of gonococcal antimicrobial resistance (AMR) testing.**

| **Strain Name** | **Azithromycin** | **Ciprofloxacin** | **Cefixime** | **Ceftriaxone** | **Gentamicin** | **Tetracycline** |
| --- | --- | --- | --- | --- | --- | --- |
| WHO F | 0.125 | ≤0.001 | ≤0.001 | 0.015 | 32 | 0.25 |
| WHO G | 0.25 | 0.125 | 0.007 | 0.125 | 32 | 8 |
| WHO L | 0.5 | 16 | 0.06 | 2 | 16 | 0.25 |
| WHO M | 0.5 | 1 | ≤0.001 | 0.125 | 64 | 0.25 |
| WHO O | 0.5 | 0.007 | 0.015 | 0.25 | 32 | 0.125 |

**Supplementary Table S3. The minimum inhibitory concentration (MIC in µg/mL) of gold drugs (auranofin, sodium aurothiomalate and aurothioglucose) and control antibiotics azithromycin, and ceftriaxone against 48 clinical isolates of *Neisseria gonorrhoeae*. MIC_50_ and MIC_90_ are the minimum inhibitory concentrations needed to inhibit 50% and 90% of the strains.**

| **Strain Name** | **Auranofin** | **Aurothiomalate** | **Aurothioglucose** | **Azithromycin** | **Ceftriaxone** |
| --- | --- | --- | --- | --- | --- |
| *N. gonorrhoeae* WHO F | 0.007 | 0.5 | 0.5 | 0.125 | 0.015 |
| *N. gonorrhoeae* WHO G | 0.015 | 0.25 | 0.25 | 0.25 | 0.125 |
| *N. gonorrhoeae* WHO L | 0.03 | 0.125 | 0.5 | 0.5 | 2 |
| *N. gonorrhoeae* WHO M | 0.03 | 0.25 | 1 | 0.5 | 0.125 |
| *N. gonorrhoeae* WHO O | 0.03 | 0.5 | 1 | 0.5 | 0.25 |
| *N. gonorrhoeae* 165 | 0.125 | 16 | 16 | 1 | 0.06 |
| *N. gonorrhoeae* 166 | 0.06 | 1 | 1 | 1 | 0.125 |
| *N. gonorrhoeae* 167 | 0.06 | 0.5 | 8 | 16 | 0.015 |
| *N. gonorrhoeae* 168 | 0.06 | 0.125 | 0.125 | 0.5 | 0.125 |
| *N. gonorrhoeae* 169 | 0.06 | 0.25 | 0.125 | 1 | 0.125 |
| *N. gonorrhoeae* 170 | 0.06 | 0.125 | 0.5 | 1 | 0.06 |
| *N. gonorrhoeae* 171 | 0.06 | 0.5 | 0.5 | 1 | 0.06 |
| *N. gonorrhoeae* 172 | 0.125 | 0.5 | 0.5 | 1 | 0.06 |
| *N. gonorrhoeae* 173 | 0.06 | 0.125 | 0.125 | 1 | 0.125 |
| *N. gonorrhoeae* 175 | 0.06 | 0.5 | 1 | 0.5 | 0.015 |
| *N. gonorrhoeae* 176 | 0.125 | 1 | 4 | 0.5 | 0.03 |
| *N. gonorrhoeae* 177 | 0.125 | 1 | 1 | 0.5 | 0.015 |
| *N. gonorrhoeae* 178 | 0.125 | 1 | 1 | 0.5 | 0.03 |
| *N. gonorrhoeae* 179 | 0.03 | 0.125 | 0.125 | 4 | 0.015 |
| *N. gonorrhoeae* 180 | 0.06 | 0.25 | 0.25 | 0.03 | 0.06 |
| *N. gonorrhoeae* 181 | 0.06 | 0.5 | 0.25 | 256 | 0.03 |
| *N. gonorrhoeae* 182 | 0.125 | 0.5 | 0.125 | 0.5 | 0.06 |
| *N. gonorrhoeae* 183 | 0.125 | 0.25 | 0.5 | 0.5 | 0.03 |
| *N. gonorrhoeae* 184 | 0.125 | 0.25 | 8 | 0.25 | 0.06 |
| *N. gonorrhoeae* 185 | 0.125 | 0.5 | 0.25 | 0.25 | 0.06 |
| *N. gonorrhoeae* 186 | 0.06 | 0.25 | 0.25 | 0.25 | 0.125 |
| *N. gonorrhoeae* 187 | 0.125 | 0.5 | 0.5 | 0.5 | 0.03 |
| *N. gonorrhoeae* 188 | 0.125 | 0.25 | 0.25 | 0.25 | 0.06 |
| *N. gonorrhoeae* 189 | 0.125 | 0.25 | 0.25 | 0.5 | 0.06 |
| *N. gonorrhoeae* 190 | 0.06 | 0.25 | 0.25 | 1 | 0.06 |
| *N. gonorrhoeae* 191 | 0.03 | 0.25 | 0.5 | 1 | 0.125 |
| *N. gonorrhoeae* 192 | 0.06 | 0.25 | 0.125 | 1 | 0.03 |
| *N. gonorrhoeae* 193 | 0.03 | 0.125 | 0.25 | 0.5 | 0.06 |
| *N. gonorrhoeae* 194 | 0.06 | 0.25 | 0.5 | 0.5 | 0.5 |
| *N. gonorrhoeae* 195 | 0.06 | 0.125 | 0.5 | 2 | 0.06 |
| *N. gonorrhoeae* 196 | 0.06 | 0.125 | 0.5 | 0.5 | 0.03 |
| *N. gonorrhoeae* 197 | 0.06 | 0.25 | 0.25 | 8 | 0.06 |
| *N. gonorrhoeae* 198 | 0.06 | 0.125 | 0.5 | 2 | 0.03 |
| *N. gonorrhoeae* 199 | 0.06 | 0.06 | 16 | 1 | 0.06 |
| *N. gonorrhoeae* 200 | 0.125 | 0.25 | 0.5 | 1 | 0.125 |
| *N. gonorrhoeae* 201 | 0.06 | 0.125 | 0.25 | 1 | 0.125 |
| *N. gonorrhoeae* 202 | 0.06 | 0.25 | 1 | 16 | 0.015 |
| *N. gonorrhoeae* 203 | 0.125 | 0.25 | 0.5 | 1 | 0.125 |
| *N. gonorrhoeae* 204 | 0.125 | 32 | 16 | 0.5 | 0.06 |
| *N. gonorrhoeae* 205 | 0.06 | 0.125 | 0.25 | 1 | 0.06 |
| *N. gonorrhoeae* 206 | 0.06 | 0.125 | 0.25 | 1 | 0.06 |
| *N. gonorrhoeae* 207 | 0.125 | 4 | 1 | 1 | 0.06 |
| *N. gonorrhoeae* 208 | 0.06 | 0.125 | 0.125 | 1 | 0.125 |
| *N. gonorrhoeae* 209 | 0.125 | 2 | 0.5 | 1 | 0.03 |
| *N. gonorrhoeae* 210 | 0.06 | 0.5 | 0.25 | 1 | 0.06 |
| *N. gonorrhoeae* 211 | 0.125 | 0.25 | 0.5 | 1 | 0.06 |
| *N. gonorrhoeae* 213 | 0.125 | 1 | 0.5 | 0.5 | 0.06 |
| *N. gonorrhoeae* 214 | 0.125 | 0.5 | 0.5 | 0.5 | 0.125 |
| **MIC_50_** | **0.06** | **0.25** | **0.5** | **1** | **0.03** |
| **MIC_90_** | **0.125** | **1** | **8** | **4** | **0.06** |

**Supplementary Table S4. Spontaneous mutation frequency of auranofin at 10 ×MIC against three strains of *N. gonorrhoeae*.**

| **Drug** | **Frequency of spontaneous mutation** | |
| --- | --- | --- |
| **Strain** | **Auranofin** | **Rifampin** |
| *N. gonorrhoeae 197* | <2.4 x 10^-10^ | 1.8 x 10^-6^ |
| *N. gonorrhoeae 202* | <2.4 x 10^-10^ | 4.17 x 10^-6^ |
| *N. gonorrhoeae 206* | <2.4 x 10^-10^ | 1.2 x 10^-6^ |

**Supplementary Table S5. *In vitro* post-antibiotic effect (PAE) of auranofin and azithromycin against four strains of *N. gonorrhoeae.***

| **Drug** | **Post antibiotic effect (hours)** | |
| --- | --- | --- |
| **Strain** | **Auranofin** | **Azithromycin** |
| *N. gonorrhoeae* 181 | 10 | 8 |
| *N. gonorrhoeae* 194 | 10 | 8 |
| *N. gonorrhoeae* 186 | 10 | 8 |
| *N. gonorrhoeae* 198 | 10 | 8 |

**Supplementary Figure S6. Toxicity analysis of gold compounds against human endocervical cells (End1/E6E7).** Percentage viability of End1/E6E7 cells was measured as average absorbance ratio (test drugs relative to DMSO) for cytotoxicity analysis for all three gold compounds (in triplicates) at 8×, 4×, 2×, and 1× MIC against End1/E6E7 cells using MTS 3-(4,5-Dimethylthiazol-2-yl)-5-(3-carboxymethoxyphenyl)-2-(4-sulfophenyl)-2H-tetrazolium.
